# Prospects and Pitfalls of Machine Learning in Nutritional Epidemiology

**DOI:** 10.3390/nu14091705

**Published:** 2022-04-20

**Authors:** Stefania Russo, Stefano Bonassi

**Affiliations:** 1 EcoVision Lab, Photogrammetry and Remote Sensing Group, ETH Zürich, 8092 Zurich, Switzerland; 2 Department of Human Sciences and Quality of Life Promotion, San Raffaele University, 00166 Rome, Italy; stefano.bonassi@sanraffaele.it; 3 Unit of Clinical and Molecular Epidemiology, IRCCS San Raffaele Roma, 00163 Rome, Italy

**Keywords:** nutritional epidemiology, artificial intelligence, machine learning, modelling

## Abstract

Nutritional epidemiology employs observational data to discover associations between diet and disease risk. However, existing analytic methods of dietary data are often sub-optimal, with limited incorporation and analysis of the correlations between the studied variables and nonlinear behaviours in the data. Machine learning (ML) is an area of artificial intelligence that has the potential to improve modelling of nonlinear associations and confounding which are found in nutritional data. These opportunities notwithstanding, the applications of ML in nutritional epidemiology must be approached cautiously to safeguard the scientific quality of the results and provide accurate interpretations. Given the complex scenario around ML, judicious application of such tools is necessary to offer nutritional epidemiology a novel analytical resource for dietary measurement and assessment and a tool to model the complexity of dietary intake and its relation to health. This work describes the applications of ML in nutritional epidemiology and provides guidelines to avoid common pitfalls encountered in applying predictive statistical models to nutritional data. Furthermore, it helps unfamiliar readers better assess the significance of their results and provides new possible future directions in the field of ML in nutritional epidemiology.

## 1. Introduction

The ever increasing hardware and Internet accessibility and the novel big data sources are just some of the key elements that are now enabling technologies to unlock the power of artificial intelligence (AI) and machine learning (ML). ML is an area of AI that works by learning from data. Here, analytical model building is automated to provide predictions without being explicitly programmed to do so. Therefore, it does not come as a surprise that ML methods are becoming progressively pervasive in our society, where most fields of science have undergone a big data revolution.

Nutritional epidemiology uses dietary analysis to study the complex link between nutritional intake and health. Given the ongoing technology revolution resulting in an increased amount of available electronic data, nutritional epidemiology research has recently been seeing a rapid expansion of ML applications, especially in the field of data fusion, modelling of nonlinear associations and feature reduction. Nevertheless, reconciling between the well established scientific principles of nutritional epidemiology and this novel field is proving to be difficult.

Given the still young state of ML in nutritional epidemiology, the goal of this work is to provide a critical overview of the applications of ML in nutritional epidemiology research, its limitations and future perspectives. Clearly, this demands an ever deeper understanding of such approaches at hand. Many researchers have addressed this problem by teaming up with or contracting mathematically qualified scientists, but the shortage of suitably trained professionals means that research groups often rely on untrained or inexperienced scientists to perform complex data analysis. Although previous commentaries have offered recommendations and guidelines to avoid common pitfalls associated with these analysis tasks, in practice, following these guidelines has proven more difficult than expected. Furthermore, these frameworks have never been provided for the field of nutritional epidemiology. By bringing in a novel perspective on some of the key statistical issues, we hope that the exposition below will help readers better apply such techniques and interpret results from studies. In the next sections, we will first review current areas of research and limitations of nutritional epidemiology. Following that, we provide the introductory concepts and a literature review on ML and big data. We then describe the current approaches of ML in nutritional epidemiology and focus on its benefits, limitations and common pitfalls to avoid when applying data-driven models to the field of nutritional epidemiology. We conclude by proposing future directions for research on the applications of ML in nutritional epidemiology.

## 2. Nutritional Epidemiology

Nutritional epidemiology is a discipline that studies the impact of diet and/or nutritional elements on disease occurrence in populations. The findings from nutritional epidemiology are generally applied for creating dietary policies, food fortification interventions, restriction of substances, or recommendations for prevention of cancer, chronic diseases, obesity and congenital malformations (Satija et al. [1]).

To do so, nutritional epidemiology consists of three main research areas (Illner et al. [2]): (1) exposure and outcome measurement (e.g., nutritional intake and disease occurrence) through data collection on large groups of participants; (2) choice of the study design; and (3) analytical and statistical techniques to assess the magnitude of the association between these two measures. Nutritional epidemiology is characterised by uncertainty and an incredible number of factors that can play a role in the measured association. Therefore, the process of causal inference is complex, and it is generally rare for a causal relationship to be considered unequivocal. The major challenges in the process of causal inference are described below.

### 2.1. Errors in Measurement Methods

Sources of error in dietary assessments can be divided in two categories (Thornton and Villamor [3]): *random errors* are generally unintentional mistakes such as marking the wrong frequency column, or copying a wrong number when switching from a paper to an electronic form. Such errors are considered noise in the sense that they cover the real signal, i.e., the association between cause and effect. Plausibility checks can often be employed to discover such errors in a dataset. The effects from random errors can be mitigated by increasing the amount of data collected. The second types of errors are the *systematic errors* which refer to biases across the population and mainly depend on the patient. These include memory problems, lack of correct estimation in the frequency of foods consumed and portion size (under or over-reporting of intake) and social desirability bias. The latter describes a situation in which the interviewees report the more desirable or socially accepted response (Hebert et al. [4]). In Section 4.1, we will describe how ML can be used to improve the accuracy of dietary records.

### 2.2. Nonlinearities

The simplest models assume a linear association between exposure and outcome. However, most of the times a nonlinear relationship exists. A variety of approaches have been developed for dealing with nonlinearities, but they are usually ad hoc solutions based on experience, needing domain expertise and careful assessment and interpretation of the results. Some factors are specifically crucial in jeopardising the detection of nonlinear effects, which in turn cause misinterpretation of the results (May and Bigelow [5]). Some of these factors are: (i) sample size: If the data are not large enough, detecting the true relationship between exposure and outcome is troublesome. Additionally, if the range of exposure is small, any inference of nonlinearity can pass unnoticed, together with possible threshold effects; (ii): Individual susceptibility is an important factor that can contribute to a disease. In this case, adding data from genetic studies could be helpful although laborious and challenging to realise; (iii): wrong models: Sometimes, authors just do not use the right model to estimate the true function between exposure and outcome. A quick fix for such a problems is to approach the modelling process by using multiple approaches in an exploratory phase until the right model is found. For this reason, results from a specific study should be judged in the context of their specific application. In Section 3 and Section 4.2.1 we will describe how complex ML models have been shown to deal particularly well with nonlinear data.

### 2.3. Confounding

Confounding is one of the main sources of systematic errors that can appear whenever assessing causality in epidemiologic studies (Greenland and Morgenstern [6]). Confounding represents an alternative explanation for the association between the exposure and the outcome, and it is introduced by a third variable called a confounder. Confounding is a critical error in nutritional epidemiology. For it to occur, the confounder must be associated, but not measured, with both the exposure and the outcome. In this case, statistical adjustment in analyses can be performed to control for some of the effects of confounding. One example is entering confounders as covariates in analytic models (Zeraatkar et al. [7]). However, complete removal of the influence of confounding cannot be assured. ML approaches to mitigate confounding are described in Section 4.3.

### 2.4. Missing Data

After an epidemiologic study, data that were collected are then recorded and inserted into a database. Here, it is crucial to consider which information was not provided by the interviewees, that is, missing data. There are different techniques that deal with missing data (Sangra and Codina [8]), the most common being: (i) listwise method, which completely deletes the interviewee presenting missing data, however it affects the power of the tests; (ii): pairwise deletion, which keeps the subject whenever such missing values do not affect the analysis, otherwise entirely deletes a subject; (iii) imputation, which replaces missing values with the mean of other values, or though regression, etc. This approach can however introduce random bias in the later modelling stage, which is not taken into account by current methods. Similar to the errors related to measurement methods, missing data can be imputed with ML models or can be dealt with during training of a ML model, as explained in Section 5.

An exciting area of future research regards ML, which has the promise of mitigating or solving some of these challenges. ML is presented in the Section 3.

## 3. Machine Learning

ML supports domain experts by automatically learning from data, thus removing the need for manual analytical model building. Such techniques are more flexible than the classical statistical model approaches (Ciavatta et al. [9]) because they can take advantage of data-rich applications. ML models are then integrated into different downstream tasks and service applications to provide data insights and support decision making. Clearly, data are an essential component of ML. The first 2 decades of the 21st century have witnessed a sharp increase in the volume of digitally available data, which in turn has allowed the development and further deployment of ML in a wide range of applications in healthcare, finTech, cybersecurity, robotics, predictive maintenance to improve processes, customer experiences, and decision outcomes. The topic of digital data correlates with big data, a terminology that is usually found in the AI field. Such a term refers to data coming in large amounts. An enabling technology for big data has been the ever increasing amount of low-cost smaller electronics, computing devices, sensors, and the Internet of Things (IoT). IoT can be considered as a system of such physical devices connected through an area network and communicating with each other. An IoT device can also be a smartphone. This interaction can generate an enormous quantity of data, which in turn can be used to create new services and applications through ML (Shanthamallu et al. [10], Mahdavinejad et al. [11]). In healthcare applications, IoT and big data analytics through ML is now allowing applications which facilitate the collection and smart usage of patients’ data, improving treatments and services. This can also be used for population healthcare projects and clinical research.

### 3.1. Training and Evaluation of Machine Learning Models

The main goal of using ML is to produce a model that enables the prediction of a value $\hat{y}$ for any observation represented as a *d*-dimensional input vector x which has the values for *d* variables (features). Model training in ML is referred to as the process by which a mathematical model ϕ is identified by means of data selected for this task. During model training, the model’s parameters are identified based on the training dataset (Hastie et al. [12]). For ML models to understand how to provide predictions, the training datasets are fed into the ML algorithm; this is then followed by validation datasets which ensure that the model is interpreting this data accurately and it is not *overfitting*. Overfitting happens whenever a ML model learns how to exactly reproduce the pattern of the training data. When this happens, the algorithm unfortunately cannot generalise well on new, unseen data. Broadly speaking, the more data are provided to the ML system, the better that model can learn to generalise and improve its performance. Model performance is generally defined as the model’s generalisation capacity, i.e., the ability of a training model to correctly predict new values for previously unseen data points. The data can be represented in different ways: (i) We can use a collection of observations, each describing a state *y*. For example, we can have different biomarker measurements for the same patient which might express that observation as belonging to the class *y* denoting cancer. (ii) Time-series data representing the temporal changes of the variables under study might be used. These data are usually used for regression problems, where the goal is to predict what the next values will be, given the previous ones. (iii) Image data, mainly used recently in computer vision applications, might also be employed.

### 3.2. Machine-Learning Techniques

Based on the availability of target labels, ML models can be broadly divided in supervised and unsupervised models.

Supervised models are trained using the training data with associated target labels $y_{i}$ (Caruana and Niculescu-Mizil [13]). The model ϕ is trained with the given dataset $D={\left\{\left(x_{i},y_{i}\right)\right\}}_{i=1}^{N}$ to predict the associated label. These predictions are compared with the target labels, and the parameters of the model are learned by means of the loss function and the optimisation algorithm. The loss function evaluates how well the model is fitting on the given data. The optimisation algorithm is used to find the values of the model’s parameters that minimise the loss function. Most typically, the labels are provided by a human expert. After training, the models can be used to predict the target label for each new unseen data point. In *unsupervised techniques*, the model scores the data solely based on the patterns in the training dataset without any target label (Hastie et al. [14]). In this case, the training dataset $D={\left\{x_{i}\right\}}_{i=1}^{N}$ consists of *N* data points where (i=1,…, N). ML techniques can also be divided in *classification* applications, where $y_{i}$ can only acquire discrete label values associated to a class. A class denotes a set of data having common characteristics. *Regression* problems involve learning the underlying function f(x) of input–output, which means predicting a continuous value $y_{i}$.

### 3.3. Neural Networks and Deep Learning

Between the most employed ML models, special mention goes to artificial neural networks (ANNs) due to their vast usage (Hassoun et al. [15]). ANNs compute the predicted output value by means of a network of simple yet nonlinear unit operations (neurons). Each neuron has its own set of parameters. While ANNs are essentially nonlinear regression models, they are extremely flexible due to the ability to string an almost arbitrary number of neurons together, specifically by using large numbers of layers between data samples and predicted labels (hidden layers), each including many neurons. ANNs are used in both supervised regression and classification tasks. A deep neural network (DNN) (LeCun et al. [16]) falls under the deep learning umbrella and can be considered as multiple ANNs composed of several layers that have the ability of learning very complex functions. In fact, in the mathematical theory of ANNs, the universal approximation theorem (Winkler and Le [17]) states that ANNs and specifically DNNS, have the capabilities of universal approximators, i.e., no matter what underlying function represents the data, there is always a network that can approximately approach the result. Of course, this is an ideal situation that depends on the architectural choice and on the quality (and quantity) of input data. Clearly, this can be proven to be very useful in modelling complex diet–disease processes. There are different types of DNNs, and their main difference lies in the types of neurons (nodes) used (LeCun et al. [16]). For example, convolutional neural networks (CNNs) are mostly used for image recognition and for their ability to learn hierarchical feature representations. Recurrent neural networks are also another type of DNNs, where the connections between nodes form a graph along a temporal sequence and are mainly using for time-series data and speech recognition.

To conclude this section, we summarise in Table 1 the different learning types, tasks and ML models that are most commonly found in the literature. Note that some ML models can be used for more than one technique (e.g., ANNs can be applied in both classification and regression tasks). For a deeper dive into these, we refer the reader to Hastie et al. [14], LeCun et al. [16], Murphy [18].

## 4. Applications and Common Pitfalls

In this section, we describe different applications of ML in the field of nutritional epidemiology, highlighting their strengths and weaknesses.

### 4.1. Health and Dietary Input Data

#### 4.1.1. Increasing the Amount of Data

Nutritional digital data can be generated through multiple means, thanks to the ubiquity of Internet-connected computers and smartphones. For example, ML models can now successfully leverage the entire contents of the electronic health records (Morgenstern et al. [19]). In wearable technology (Phillips et al. [20]), dietary assessment can be conducted by using wearable devices containing gyroscopes and/or accelerometers. These can track wrist movements and indirectly record lifting of hands and cutlery as an approximation to account for calorie intake and eating patterns (Vu et al. [21]). Of course, such systems can also be used to monitor daily physical activity. Wearable sensors can also be used to continuously monitor glucose (Cappon et al. [22]) for diabetes treatment and self-management in downstream ML-based decision-support systems (Contreras et al. [23], Kavakiotis et al. [24]).

Additionally, mobile calorie counting apps (Limketkai et al. [25]) can record large amounts of data from the users, self-reporting daily portions and calorie intakes. Nowadays, it is estimated that there exist about 165,000 publicly available mobile health apps related to health and wellness (Kao and Liebovitz [26]). Such systems can represent a large opportunity for nutritional epidemiologic studies since the data does not need to be collected inside the clinics. Rather, it can be collected remotely at home and on the go, without suffering from recollection bias. Furthermore, compared to traditional data collection methods (e.g., phone interviews etc.), mobile self-reporting is also less time demanding. A simple and inexpensive yet powerful way to track dietary habits is through grocery purchases in smartphone-tracking applications. These records have been used in several public health nutrition research studies to evaluate interventions (Bandy et al. [27]). Another way to decrease the burdensome task of collecting dietary data is to use ML-based natural language processing techniques, which convert speech into text or input variables, e.g., by using a smartwatch combined with an audio-based detector of chews and swallows to identify eating behaviours (Kalantarian and Sarrafzadeh [28]). Dietary data can also be automatically “mined” through ML approaches from social media for ecological studies to study community-level health outcomes (Shah et al. [29]). In another study (Gerina et al. [30]), a ML model was used to detect cooking activities based on air quality sensor data. The model’s output was integrated with a social robot that, by interacting with the participant, supported filling in a food diary.

Such approaches can facilitate more longitudinal, repeated dietary measurements, allowing for expanded sample sizes thereby increasing statistical power. However, as sample size grows, it decreases the quality of data aggregated through wearable devices, apps or social media due to the lack of supervision by a clinical researcher during collection.

#### 4.1.2. Improving Data Quality

By its very nature, a ML model is sensitive to the quality of the data used during its training and validation. The rule of “*garbage in, garbage out*” (Grimes [31]) applies here, where even small errors or bias in the training data can lead to unexpected consequences in the model’s prediction.

To increase and improve the accuracy of self-reported dietary records during food logging, an active area of research has recently seen ML and deep learning models used to classify foods and calories from pictures of users (Lo et al. [32], Tay et al. [33], Sahoo et al. [34], Lo et al. [35]). Accuracy was reported to be as high as 90%. Another approach is to evaluate food sizes (Ege et al. [36]) from food images and use the resulting value to evaluate caloric intake. Such approaches can be applied to address systematic and random errors. However, image-based dietary assessment relies heavily on computing algorithms and storage facilities, which can greatly increase the processing time. For example in (Puri et al. [37]), the authors report an image-based assessment time of 33 s. Furthermore, such analysis can be troublesome if the dataset to train (and test) the model is limited, such as in (Zhu et al. [38], Woo et al. [39]), which could make it necessary to semi-automate the process of food recognition, as presented in (Jia et al. [40]). To mitigate this issue, large datasets of food images are becoming increasingly available (Min et al. [41], Aguilar et al. [42]). The idea here is that deep learning models for image recognition such as CNNs can be pretrained on such large datasets and then, thanks to a technique called transfer learning, can be used for online applications in a continuously learning manner (He and Zhu [43]), further decreasing prediction times.

Low quality input data can also lead to a biased ML model. These raise ethical questions whether we can safely trust decisions taken based on ML models’ predictions. In fact, it is critical to ensure that the data is representative of the population under study and not skewed or imbalanced. Large amounts of data would usually suffice to prevent biases of the ML model, but even large datasets need to be of high quality. For example, patients with a low socioeconomic status usually have limited access to healthcare facilities (Arpey et al. [44]). They therefore have insufficient information in their electronic health records, leading to a biased ML model presenting disparities (Gianfrancesco et al. [45]). A possible solution to avoid such bias is to make sure that features that are biased, such as ethnicity and social determinants of health, are also included in algorithms (Gianfrancesco et al. [45]). Related to model bias is model transparency (i.e., *why* a certain prediction was given) which is necessary for real-world implementations since special care needs to be take whenever the ML model will be used for downstream tasks such as patient care. We will discuss the aspects of transparency (interpretability and explainability) in Section 5.4.

### 4.2. Modelling of Dietary Variables

#### 4.2.1. Non-Linearities

The diet–disease relationship in epidemiologic research is mostly conducted in its simplest form by considering linear models of association between exposure and outcome (Boeing [46]). However, this assumed relationship is generally not correct as nonlinear relations exist (Ioannidis [47]). For instance, several studies related to cardiovascular diseases have shown that salt, carbohydrates and fats possibly present a U- or J-shaped relation with such disease (Kong et al. [48], Investigators et al. [49]), largely due to confounding factors. As a consequence, if incorrect linear assumptions are taken during the modelling phase, this can cause spurious associations and biased effect estimates (Bodnar et al. [50]).

Complex ML and deep learning algorithms such as ANNs and DNNs can overcome these limitations due to their ability to model complex relationships between input variables. For example, in (de Cos Juez et al. [51]), an ANN was implemented to relate 38 diet and lifestyle variables to bone mineral density in post-menopausal women. The sample size, however, was relatively small, with only 200 patients. No information was given about using a validation dataset to prevent overfitting of the model. In a cross-sectional survey (Zeng et al. [52]), the risk of hyperuricemia based on dietary information was estimated using an ANN. In (Chew [53]) DNNs were used to classify images of patients with age-related macular degeneration supporting the authors in evaluating the importance of nutritional supplements. Other examples of DNNs for modelling nonlinear diet–disease relationships are still scarce in the nutritional epidemiology literature. We suspect this is due to the generally low availability of large datasets. In fact, in other broader disciplines such us clinical and epidemiologic research, where data availability is improved, we found that several studies employ DNNs, e.g., a deep learning model for the detection of breast cancer (Puvanesarajah et al. [54]); a CNN model for non-imaging diagnostic to predict for skin cancer (Vivot et al. [55]); or for diabetic retinopathy screening (Wong and Bressler [56]). In medical and epidemiologic research, it is also reported that DNNs perform better than ML approaches (Byeon [57], Xiong et al. [58], VoPham et al. [59]) due to their exceptional capabilities to model nonlinear relationships and because they need virtually no feature engineering of the input data. Because of this, we believe that a large amount of structured data is still needed for nutritional epidemiology to unlock the true power of DNN models.

#### 4.2.2. Dimensionality Reduction

Curse of dimensionality refers to a phenomenon that arises when data in high-dimensional spaces (i.e., with a high number of variables/features) are used for analysis or modelling purposes. Given the large amount of possible explanatory variables, modelling studies in nutritional epidemiology have become difficult to conduct, and the task of identifying the most predictive ones is extremely challenging (Bodnar et al. [50]). In addition, common statistical models cannot easily deal with a large number of variables. In this case, ML can be used as a dimensionality reduction technique. Such techniques can be split into (1) feature extraction and (2) feature selection methods.

*Feature extraction* works by finding a combination of new features from the original ones. Such algorithms simplify modelling, thereby partially overcoming the curse of dimensionality. Depending on the technique that is used, these methods find the best linear or nonlinear transformation that reduces the number of dimensions with a minimum loss of information. Examples of linear methods are principal component analysis (PCA) and linear discriminant analysis. In (Hoffmann et al. [60]), three linear methods (PCA, reduced rank regression and partial least squares) were employed for feature extraction to derive dietary patterns from 49 food groups related to type 2 diabetes. The authors in (Zhang et al. [61]), used PCA to derive dietary patterns predictive of cardiovascular disease risk. In (Santos et al. [62]), PCA was applied on 34 variables expressing the mean food intake of 1102 individuals from a population-based study. Nonlinear dimensionality reduction techniques are deep autoencoders and manifold learning (Morgenstern et al. [19]). Deep autoencoders are especially interesting since they are DNNs and combine the advantages of being both unsupervised and nonlinear approaches, allowing for the embedding of data into a low-dimensional representation while conserving its properties (Falissard et al. [63], Wang et al. [64]). Clustering techniques (e.g., Gaussian mixture models or k-means clustering) can also be included into feature extraction techniques as they can eliminate noisy variables. For example, (Kwon et al. [65]) employed k-means clustering to pinpoint risk factors for low muscle mass based on nutritional factors. The algorithm generated clusters of patients based on their dietary and health-related data, where patients within the same cluster had similar attributes. ML logistic regression was then applied to find risk factors in each cluster.

*Feature selection* methods aim at reducing the dimensionality in a large dataset. Rather than transforming or grouping the variables into a new representation, feature selection methods can restrict the number of variables to a smaller subset by applying a selective filter. The selected variables are chosen based on how informative they are for the prediction of the outcome (Walter and Tiemeier [66]). For example, techniques such as permutation feature importance (Altmann et al. [67]) can be used to train the ML model recursively with different variables, thereby finding the ones providing best model performance. In (Zeevi et al. [68]), this technique was used to find the most important variable related to glycemic responses in a regression algorithm, which was found to be related to microbiota. In (Dipnall et al. [69]), a methodology for feature selection using ML on a large epidemiological dataset was successfully implemented for detecting 3 out of 67 biomarkers (red cell distribution width, serum glucose and total bilirubin) associated with depression.

The main downside of such dimensionality reduction approaches is that they are usually difficult to automate and apply to a different dataset because of their complexity and application-specific fine-tuning. Additionally, they require extensive domain expertise, experience, and process understanding (Russo et al. [70]).

### 4.3. ML Approaches to Confounding

In nutritional epidemiologic studies, confounding and multicollinearity are systematic errors that can cause misinterpretation of the results. That is because they generate a false relationship between the dietary variable and the disease risk. This aspect is particular challenging in nutritional data since nutrients from different foods are not only correlated with each other but also with different outcomes (diseases) (Trepanowski and Ioannidis [71]). Confounding is usually controlled by a statistical adjustment during data analyses. This approach, however, requires domain confidence and expertise and making assumptions about the measured variables.

A way to deal with confounding through ML is by creating “high-capacity models”. In ML, capacity is a term representing model complexity, i.e., a model with higher capacity is expected to be able to model more complex relationships between the variables. Usually, high-capacity models are ANNs or deep-learning models presenting an extreme number of parameters (in terms of millions). Training high-capacity deep-learning models with a high number of observations and variables has been shown to better deal with confounding in clinical research (Brisk et al. [72], Badgeley et al. [73]). That is because such models have the capability of processing data through their several layers and create entirely new types of variables. This approach not only adjusts for confounding, but also for any existing nonlinearity (Morgenstern et al. [19]) by taking advantage of the data richness in number of observations and variables. High-capacity models of this type however are prone to overfitting and errors in the testing phase. In the next section we will discuss the issue of overfitting together with different methods used to adjust for it.

## 5. Practical Recommendations

### 5.1. Data Preparation

The most important step after data—especially *big* data—collection, is to preprocess (i.e., clean and/or put in the right format), store, and make it available for research (García et al. [74]). Additionally, metadata should also be provided. Finally, each of the above data preparation steps should also be well documented, ideally providing the end user with all the versions of the data, from raw to clean. Addressing the points above can be challenging and time demanding, but it is fundamental. In fact, the success of ML techniques is dependent on the quality of the data that they operate on (Kotsiantis et al. [75]).

At this stage, missing data should be handled correctly. Imputation of missing data through ML techniques has been extensively studied (Lakshminarayan et al. [76], Richman et al. [77], Batista and Monard [78]) and has shown to outperform common imputation statistical methods (Jerez et al. [79]). Different ML algorithms can be applied, such as k-nearest neighbours and self-organisation maps. Recently, nonlinear techniques, such as ANNs, are also employed (Al-Milli and Almobaideen [80]).

Dimensionality reduction techniques and feature engineering are also part of this stage. Feature engineering is the process of adding or creating new features with the goal of supporting and increasing the information provided to the ML model (Heaton [81]). Such a step is usually cumbersome and requires domain expertise. For example, in a study exploring the predictive power of nutrients for cardiovascular disease using ML (Morgenstern et al. [82]), the authors combined features concerning cultural and racial origin and household income, and derived variables based on smoking as well as on the participants’ immigrant status, age, and years since immigration. Compared to ML algorithms, an advantage of deep learning models is that they do not require feature engineering due to their abilities to extract meaningful information from data automatically (LeCun et al. [16]).

During data preparation, labelling is also conducted. This is the process of assigning a target to each observation. Data labelling is necessary for a supervised ML model to learn from the training dataset to predict the associated label (target). Labels are also needed in both the supervised and unsupervised case for performance evaluation (Russo et al. [83]). While labelling an entire dataset is tedious work that requires a dedicated team of experts, correct data labelling is crucial for any ML model. For these reasons, repeated-labelling strategies can been employed (Sheng et al. [84]). For example, a minimum of two people is required to label the data, and target labels are then computed by merging the provided results to avoid bias due to decision fatigue. Additionally, a user interface designer is needed to provide a simple, intuitive system to label data.

### 5.2. Data Quality and Quantity

“How much data is enough for a ML model”? There is no correct answer to this question, as it usually depends on the type of model chosen; the number of variables (the higher the dimension of the dataset, the more observations are needed for the model to create an input–output function); and the data quality (Gudivada et al. [85]). Data quality refers to the presence of noise and missing data, but it also deals with how representative of the population under study it is. Unfortunately, the presence of low-quality observations in the data will negatively impact any ML method. A way to address such problems is through specialised loss functions (Wang et al. [86]) which reduce the weights of low-quality (or imputed) observations during training so that the model focuses more on the most informative samples. For example, in (Tran et al. [87]) a focal loss is employed for improving early detection and classification of pulmonary nodules.

On the other hand, if the only limitation is the amount of data (i.e., data shows a signal-to-noise ratio good enough to learn meaningful input–output relationships), techniques such as regularisation and k-fold cross-validation can be used to support correct training of ML models. We will discuss these techniques below.

### 5.3. Avoiding Overfitting

Overfitting is most probably one of the main errors that happen when training a ML model. Common causes of overfitting are related to the quantity of data (the fewer the data, the less the model generalises well) and using a model that is too complex (we mentioned before the term *high capacity*). As a general rule, we are in the presence of overfitting whenever the performance of a trained ML model on an unseen test set is considerably lower than the training set. In general, we should beware of results that are too good to be true.

A method used to monitor the correct training of ML models is k-fold cross-validation (Rodriguez et al. [88]). This is a technique that uses *k* numbers of held-out validation sets to constantly evaluate whether the model is overfitting on the training data during the training process. Such a technique is especially convenient for small datasets (which is usually the case for nutritional epidemiology data). In this process, the model is tested during training on an unseen data set. If the performance of the model on the held-out set is much worse than the ones on the training data, it means the model is overfitting and: (i) training should be stopped earlier; or (ii) regularisation techniques should be used.

Regularisation is one of the most important concepts of ML and is a technique that prevents the model from overfitting by discouraging learning a more complex or flexible model (Hastie et al. [12]). Regularisation comes into play during model training, i.e., by shrinking the coefficient estimates towards zero or by preventing them from rising too high.

### 5.4. Dealing with Biased Data

ML models are used to generate predictions which are then implemented in downstream tasks such as diagnosis, causality assessment, as well as decision making in self-driving cars or the automation industry. There are some instances, however, where an ML-based prediction could be incorrect and cannot be relied upon because the predictions are biased towards a particular class.

In fact, while it is nearly impossible to point out why a particular decision was made because ML models are considered “black boxes”, a large field of research focuses on interpretable and explainable AI which aims at providing model transparency (Holzinger et al. [89], Gunning et al. [90]). Specifically, interpretability is about understanding the cause and effect within the ML system, that is, how much are we able to predict what is going to happen, given an input. Explainability, on the other hand, is the extent to which the internal architecture and mechanics of a ML model can be explained in human terms. Another approach also focuses on what has been called Bayesian deep learning (Kendall and Gal [91]), which aims at obtaining a realistic, well-calibrated expression of when and how much a model is uncertain about its own prediction and therefore how trustworthy such a prediction is. A Bayesian neural network employs a prior distribution for each weight of a neural network, then posterior inference is applied. Interpretability and explainability in ML are still an open research question, and special attention should be paid to how much a ML model’s predictions are trusted to make final decisions.

### 5.5. Performance Metrics

Whenever a ML model is used during training or testing, it is critical to evaluate its predictive performance. For regression tasks, these are usually measured in terms of $R^{2}$ or explained variance, while classification tasks generally require an accuracy metric, that is:

(1) $$\mathrm{Accuracy}=\frac{tp+tn}{tp+tn+fp+fn},$$

where true positives (*tp*) are the number of correctly identified members of the disease class, true negatives (*tn*) the number of correctly classified members of the no-disease class, false negatives (*fn*) and false positives (*fp*) are, respectively, the number of incorrectly classified no-disease and disease (this equation refers to a case with two classes only). Clearly, an accuracy close to one represents a ML model with powerful predictive capabilities. However, the accuracy metric works best only when the two classes (no-disease and disease) are balanced, which is usually not the case. Generally in fact, the data can be skewed towards the no-disease class which will result in a prediction bias towards the majority class. In this case, the accuracy metric will still return a value close to one, but the model is actually unable to correctly predict the patients with disease. This will cause misleading results and incorrect conclusions. For example, in (Batterham et al. [92]) the authors employed and compared different ML models to classify participants achieving at least 10,000 steps per day in a nutrition-related intervention study. A metric called area under the curve AUC was used for performance assessment. However, the number of participants reaching the target steps was 79%, resulting in an imbalanced dataset, where such a performance metric is not ideal (for example, precision–recall curves or a F1 score would have been more feasible).

Therefore, whenever the data present imbalanced classes, it is recommended to compute the *F*1-score as:

(2) $$F1\;\;=2\;\frac{precision\cdot recall}{precision+recall},$$

where

(3) $$\begin{array}{ccc} precision=\frac{tp}{tp+fp} & \; & recall=\frac{tp}{tp+fn} \end{array}$$

Generally, there is an inverse relationship between *precision* and *recall* (recall is also sometimes called sensitivity): as one increases, the other decreases. This is called the precision–recall trade-off. While *recall* expresses the ability to find all relevant cases in the dataset (what proportion of actual diseases were identified correctly), *precision* shows the proportion of the data points the model identifies as diseases were real (e.g., a model that produces no false positives has a *precision* =1) (Davis and Goadrich [93]). In healthcare applications, *recall* is usually preferred over *precision* as it is more important to identify the disease class at the cost of having a higher number of false positives.

### 5.6. Skilled Personnel

Finally, applying ML in nutritional epidemiology needs teams of experts from a large variety of disciplines. That is because best practices and approaches need to be followed to build a productive ML team (Schelter et al. [94]). Necessary figures include: (1) a data engineer responsible for building data pipelines architectures and infrastructure; (2) a data scientist to identify cases that can be solved with ML and develop custom ML models; (3) a ML scientist/researcher to optimise and deploy models to production and conduct research for novel ML use cases and applications. Building such a team in small enterprises or for short-term research projects, however, is a challenge and can lead to lack of adequate personnel and scientific credibility of conducted research. Usage of consultancy firms can be helpful, although expensive, in this case.

## 6. Conclusions

### 6.1. Critical Points for the Application of ML

This study tried to reveal current and possible future applications of machine learning (ML) to the field of nutritional epidemiology. The current literature is, however, still almost devoid of practical and successful examples of ML in this sector. We have identified some critical points that need to be addressed for future integration and development of these fields (Diebolt et al. [95]).

- Most of the studies in the literature are limited to few models and small datasets, therefore not showing the real advantages of one method compared to another. Systematic comparisons and benchmark datasets are therefore needed.
- It is important to take advantage of the datasets already collected in different studies. That means an organised system of aggregation of the data is essential, together with a regulatory framework for ensuring data privacy and trustworthiness. Extensive work is needed to ensure that research projects collect and publish datasets in a well-organised manner and with robust security.
- In addition, availability of technical skills in the use of ML, as well as access to high-performance computing, is needed to produce clear, quantifiable demonstrations of the benefits of ML in nutritional epidemiology research. This can be reached thanks to collaborations and large investments in the training of personnel and infrastructures.
- Whenever dealing with data with a low signal to noise ratio, such as survival rate or readmission rate in hospitals, several epidemiologic studies have shown that ML algorithms provide improved performance compared with traditional statistical models (Feng et al. [96], Mortazavi et al. [97]). On the other hand, the situation is overturned for data with higher signal to noise ratio, such as risk prediction of major chronic diseases or depression. In this case, ML models have been surpassed by conventional statistical models (Nusinovici et al. [98], Gravesteijn et al. [99]). We expect a similar situation to occur in nutritional epidemiology, although the high correlation between nutritional variables could also play a big role in favour of ML models.

### 6.2. Limitations of Current Work

The main challenges found while reviewing the applications of ML in nutritional epidemiology are not only the limited literature examples but also their broad range of applications. For example, studies employing deep-learning models range from modelling input–output relationships to computer vision-based food detection. To compensate for this, research papers employing ML in clinical research were also included in the present review with the goal of showing the prospective applications of ML. If that was the case, it was explicitly specified in the text.

This review focused on current applications and pitfalls of ML in nutritional epidemiology. An aspect of nutritional epidemiology which was not discussed relates to the existing biorepositories and national surveys in public health nutrition such as (Rosso et al. [100], Riboli et al. [101]). Similarly, we did not delve into the aspect of specialised medicine and nutrition as application fields for ML (Zeevi et al. [68]). The reason for these choices lies in the objective of this study, which is to provides guidelines to avoid common pitfalls for practitioners applying data-driven models to nutritional data, leaving the above topics outside the scope of our work.

Similar to our work is (Sak and Suchodolska [102]), where the authors explore the applications of AI in nutrients science research. Different than this review, however, the authors focused on a broad range of applications areas: biomedical nutrients, clinical nutrients and nutritional epidemiology. As for the latter, the study mainly focuses on using ML for data integration such as dietary assessment and IoT systems, without mentioning modelling capabilities and providing practical recommendations. To the best of our knowledge, our work is the first review that provides a state-of-the-art snapshot of the current literature, critically evaluating diverse results while revealing possible inconsistencies in the body of research. Additionally, this work provides for the first time practical recommendations and possible future directions in the field of ML and nutritional epidemiology.

### 6.3. Future Perspectives

We believe that in the future a complete integration of ML into the field of nutritional epidemiology might be accomplished by data-driven approaches, the likes of which are seen today in artificial neural networks and deep learning, despite the challenges discussed in this work. Such techniques not only have powerful modelling capacities, requiring minimal data preprocessing, but also have promising applications within the area of data augmentation. This is mainly due to their capability in the computer vision field, of increasingly extracting abstract features from images (Goodfellow et al. [103]), which has been already applied for estimating dietary intake from food pictures. Although few initial works on deep learning in nutritional epidemiology can be found in the body of research, to really reap the power of such techniques, larger datasets and efforts are still needed.

## Figures and Tables

**Table 1:** Summary of the main machine-learning categories.

Learning Type	Technique	Models
Supervised	Classification	Random Forest, Naïve Bayes,
		Support Vector Machine,
		k-Nearest Neighbour, ANN
	Regression	Linear Regression
		Logistic Regression
		Random Forest, ANN
Unsupervised	Feature extraction	PCA
		Deep Autoencoders
		Manifold Learning
	Clustering	Gaussian Mixture Models
		k-Means
		Deep Neural Networks
